# The Impact of COVID-19 on the Quality of Admission Clerkings on an Old Age Psychiatry Ward – an Audit

**DOI:** 10.1192/bjo.2022.486

**Published:** 2022-06-20

**Authors:** Ivan Shanley, Jaweria Faheem, Sandeep Bansal, Mahnur Khan, Hana Jeetun

**Affiliations:** Essex Partnership University NHS Foundation Trust, Chelmsford, United Kingdom

## Abstract

**Aims:**

On 11th February 2020 a novel coronavirus was named SARS-CoV-2, with the World Health Organisation announcing that the associate disease would be known as COVID-19. As doctors providing an inpatient psychiatric service, there were various changes in our daily practice secondary to the pandemic. These included reduced staffing levels due to illness, the need to wear personal protective equipment during all patient contact and high levels of anxiety surrounding transmission. We hypothesised that the resultant pressure on our service might impact the quality of admission clerkings to our ward, (a 17 bed functional Old Age Psychiatry ward), and therefore resolved to audit the data. We determined that “quality” of the clerking should be equated to completeness, i.e. the degree to which all desired information is included.

**Methods:**

Admission clerkings to the ward are to be completed on a pro forma built within the electronic patient record system (“Paris”). This pro forma is based on guidelines for the admission of patients to psychiatric inpatient units produced by the Royal College of Psychiatrists. The standard for the audit was set as 90% compliance with each individual section of the pro forma.

All admissions across three periods were extracted from the electronic record using the inbuilt reporting function. The periods were 1st April to 1st July in 2019 (pre-pandemic, n = 15), 2020 (early pandemic, n = 29) and 2021 (late pandemic, n = 22). Data were extracted manually from each admission clerking and recording anonymously on an excel spreadsheet, with either “yes” or “no” confirming or denying compliance with each domain (e.g. presenting complaint).

**Results:**

All domains showed improved compliance from 2019 to 2021 other than recording of the mental state examination which saw a 9.09% decrease (which is not statistically significant). Comparing the pandemic years, performance was better in the early pandemic in 4 domains, better in the late pandemic in 10 domains and equal in 6 domains. 4 domains demonstrated a statistically significant improvement compared to pre-pandemic, however 9 domains still fell below the 90% standard set.

**Conclusion:**

Despite the challenges posed by the COVID-19 pandemic the quality of inpatient admission clerkings has not only remained unharmed but in some domains significantly improved. Admission numbers increased during the pandemic periods, so it may represent greater familiarity with the clerking process, or perhaps a desire to make more comprehensive notes during a time of crisis. Repetition of the study post pandemic may be of value.

